# Effects of Subchronic Exposure to *N,N*-Diethyl-*m*-toluamide on Selected Biomarkers in Common Carp (*Cyprinus carpio* L.)

**DOI:** 10.1155/2014/828515

**Published:** 2014-03-30

**Authors:** Andrea Slaninova, Helena Modra, Martin Hostovsky, Eliska Sisperova, Jana Blahova, Iveta Matejova, Monika Vicenova, Martin Faldyna, Lenka Zelnickova, Frantisek Tichy, Zdenka Svobodova

**Affiliations:** ^1^Department of Veterinary Public Health and Animal Welfare, University of Veterinary and Pharmaceutical Sciences Brno, Palackého Třída 1/3, 612 42 Brno, Czech Republic; ^2^Department of Immunology, Veterinary Research Institute, Hudcova 70, 621 00 Brno, Czech Republic; ^3^Department of Anatomy, Histology and Embryology, University of Veterinary and Pharmaceutical Sciences Brno, Palackého Třída 1/3, 612 42 Brno, Czech Republic

## Abstract

DEET (*N,N*-diethyl-*m*-toluamide) is the most common active ingredient in the insect repellents commonly detected in European groundwater. The aim of this study was to investigate the effect of subchronic DEET exposure on biochemical and haematological parameters, antioxidant enzymes, including catalase, glutathione peroxidase, glutathione reductase, and glutathione S-transferase, and the amount of thiobarbituric acid reactive substances (TBARS) in common carp (*Cyprinus carpio* L.). Two specific proinflammatory and anti-inflammatory cytokine genes were selected to assess an immunological status of the fish. Fish were exposed for 28 days to three concentrations of DEET (1.0 µg/L, 0.1 mg/L, and 1.0 mg/L) where 1 µg/L is corresponding to the concentration found in the environment. DEET had a significant (*P* < 0.05) effect on increased RBC, decreased mean corpuscular volume (MCV), and mean corpuscular haemoglobin value (MCH) compared to control groups in the concentration of 1 mg/L. A significant decline (*P* < 0.05) in triacylglycerols (TAG) in plasma was found in the concentration of 1 mg/L compared to the control groups. The parameters of oxidative stress in tissues of common carp were weekly affected and immunological parameters were not affected.

## 1. Introduction

DEET (*N,N*-diethyl-*m*-toluamide) is the most common active ingredient in insect repellents used around the world due to its high efficacy against insects and arthropods bites [1–3]. DEET was produced and patented for usage in American military by the US government and registered for the general population in the 1950s [4, 5].

WHO and subsequently the US Environmental Protection Agency decided that an application of DEET-containing repellents in compliance with the instruction guidelines does not pose a health risk [6].

Behavioral and electrophysiological studies have demonstrated DEET interactions with antennal olfactory as well as gustatory receptors in insect [7–9]. Ditzen et al. [10] described DEET-dependent blockade of electrophysiological responses of olfactory sensory neurons to attractive odors in* Anopheles gambiae* and* Drosophila melanogaster*.

In addition, DEET inhibits insect acetylcholinesterase (AChE) [11] resulting in the accumulation of AChE in the synaptic cleft, which leads to a continuous stimulation of the postsynaptic neuron, finally causing the disruption of the transmission of the nerve impulse [12]. Moreover, it is unknown if the inhibition of the AChE is related to the repellency potential of DEET [11, 13, 14].

DEET is a mobile and persistent chemical which is commonly detected in aquatic environment around the world. Presence of DEET has been studied and monitored in various aquatic environments, such as drinking water, streams, open seawater, effluents from sewage plant, groundwater, treated effluent, and even drinking water treated with conventional water-treatment systems [5, 15–18]. Costanzo et al. [5] state that the concentrations of DEET in aqueous samples are ranging from 40 to 3000 ng/L worldwide, while the acute toxic concentrations for aquatic species vary between 4 and 388 mg/L [19].

The aim of this study was to assess the subchronic influence of DEET-containing formulation on common carp (*C. carpio*) through biometric, biochemical, and haematological parameters, oxidative stress markers, and selected immunological indices. The lowest tested concentrations of DEET responded to the environmental concentration.

## 2. Material and Methods

### 2.1. Experimental Design

The test was performed using two-year-old common carps (*C. carpio*) with average weight 277.1 ± 42.6 g. After one month of acclimatization to experimental conditions (light/dark: 12/12 h, a flow-through system), the fish were randomly distributed into ten tanks (volume 200 L). Three concentrations of DEET (1.0 *μ*g/L, 0.1 mg/L, and 1.0 mg/L) and two control groups were tested: one control with dilution dechlorinated water only and the second control with dilution dechlorinated water and solvent dimethyl sulfoxide (DMSO) in concentration 5 *μ*L/L). Ten fish in each group were divided into two replicates of five in each.

Concentrations of DEET were prepared from formulation Expedition 100+ (Lifemarque Ltd., UK). This formulation contains 95% of* N,N*-diethyl-*m*-toluamide and 5% of inert components. DMSO solvent was added to the formulation in the amount of 5 *μ*L/L of final solution. The duration of this subchronic toxicity test was 28 days. During the test, the condition of fish was checked twice daily and the temperature, pH, and the oxygen saturation of water were daily recorded. Water temperature in the test was 21–22°C. The dissolved oxygen concentrations were above 80–90% and pH ranged from 7.74 to 8.22. Other water quality parameters were as follows: COD_Mn_ (chemical oxygen demand) 1.4–1.9 mg/L; total ammonia 0.25–0.6 mg/L; NO_3_^−^ 40 mg/L; NO_2_^−^ 0.75–1.25 mg/L; Cl^−^ 30 mg/L; Cl^−^/N–NO_2_^−^ 78.9–130,4.

The experiment was conducted in a flow-through system, and the test solutions were changed twice a day. The concentrations of DEET did not decrease 80% of original concentrations during the experiment. The fish were fed commercial pellets at total rate of 1.5% body weight twice a day.

At the end of the experiment, individual blood samples were taken by cardiac puncture and heparinized (50 IU per mL of blood). The carps were euthanized and their body weight and length (with/without tail) were recorded. Samples of tissues, such as kidney, gills, brain, and liver (hepatopancreas), were removed and stored at −85°C until analyses.

### 2.2. Biometric Parameters

Two biometric parameters were calculated: the condition factor (CF) and the hepatosomatic index (HSI). The condition factor of each fish was calculated as CF = (body weight (g)/standard length (cm)^3^) × 100. The hepatosomatic index was calculated as HSI = liver weight (g)/body weight (g) × 100.

### 2.3. Haematological and Biochemical Profile

Haematological values, red blood cells count (RBC), white blood cells count (WBC), packed cell volume (PCV), haemoglobin (Hb), mean corpuscular volume (MCV), mean corpuscular haemoglobin value (MCH), and mean corpuscular haemoglobin concentration (MCHC), were determined according to Svobodová et al. [20]. Biochemical indices in plasma glucose, albumin, total protein, ammonium, lactate dehydrogenase (LDH), triacylglycerols (TAG), cholesterol, total calcium, inorganic phosphorus, lactate, alanine transaminase (ALT), aspartate transaminase (AST), alkaline phosphatase (ALP), and butyrylcholinesterase (ButChE) activities were determined using the biochemical analyzer Konelab 20i and commercial test kits (BioVendor, Czech Republic). To assess the ferric reducing ability of plasma samples (FRAP), the biochemical analyzer Konelab 20i was also used, according to Benzie and Strain [21] supplemented with slight modifications [22].

### 2.4. Immunological Profile

Samples of head kidney and spleen from 5 fish from 3 groups (control with DMSO and DEET in 1 *μ*g/L and 1 mg/L) were immediately stabilized with RNAlater (Qiagen) and stored at −80°C. Tissue samples free of RNAlater were then lysed in 1 mL of TRI Reagent RT (Molecular Research Center) and homogenized on MagNA Lyser (Roche) with 2.3 mm zirconia/silica beads (BioSpec Products). Total RNA was obtained using combination of 4-bromoanisole and the RNeasy Kit (Qiagen) according to the manufacturer's instruction. Extracted RNA was reversely transcribed with M-MLV reverse transcriptase (200 U) (Invitrogen) and oligo-dT primers at 37°C for 1.5 h and then stored at −20°C. cDNA diluted 5 times (0.5 *μ*L) was used in triplicate reactions in a final volume of 3 *μ*L using the QuantiTect SYBR Green PCR Kit (Qiagen). Primers (10 pmol per reaction) [23] specific for proinflammatory (TNF-*α* and IL-1*β*) and anti-inflammatory cytokine genes (TGF-*β* and IL-10) and for two candidate reference genes (40S and *β*-actin) used are shown in Table 1. Each run included a control free of template to test the assay reagents for contamination. PCR was performed on the LightCycler 480 (Roche). To test the variation of mRNA expression in samples, RefFinder tool (http://www.leonxie.com/referencegene.php) was used and *β*-actin candidate reference gene was selected for normalization of expression data in our experiment. The relative expression of a gene of interest (GOI) was calculated according to formula (1/2^Ct  (GOI))/(1/2Ct (reference gene)) [24].

### 2.5. An Activity of Detoxifying Enzymes and Values of Oxidative Stress

An activity of detoxifying enzyme (glutathione S-transferase GST) and indices of oxidative stress (glutathione reductase GR, glutathione peroxidase GPx, catalase CAT, and the amount of thiobarbituric acid reactive substances TBARS) were measured in different fish tissues (liver, kidney, gill, and brain). Tissue samples were weighed and homogenized using phosphate buffer (pH = 7.4). The homogenized samples were divided into two portions: the first one was for measuring of TBARS and the second one was centrifuged (11.000 g, 4°C, 20 min) to obtain supernatant fraction for measurement of GST, GR, GPx, and CAT activities and protein content. The enzyme activities were normalized and expressed per mg of protein content. Protein level was quantified by a spectrophotometric method using bicinchoninic acid [25]. All measurements were determined spectrophotometrically using Varioskan Flash Spectral Scanning Multimode Reader (Thermo Scientific). The GST activity was determined by measuring the conjugation of 1-chloro-2,4-dinitrobenzene with reduced glutathione at 340 nm and the activity was expressed as the nmol of the formed product per min per mg of protein [26]. The GR activity was determined by measuring of NADPH oxidation at 340 nm and expressed as the nmol of NADPH consumption per min per mg of protein [27]. The GPx activity was calculated from the rate of NADPH oxidation by the reaction with GR at 340 nm and expressed as the nmol of NADPH consumption per min per mg of protein [28]. The CAT activity was determined by measuring of H_2_O_2_ breakdown at 240 nm and it was expressed as the *μ*mol of decomposed H_2_O_2_ per min per mg of protein [29]. To evaluate the level of lipid peroxidation, the amount of malondialdehyde was measured using the TBARS method at 535 nm and the concentration was expressed as nmol of TBARS per gram of tissue wet weight [30].

### 2.6. DEET Concentration in Water

The level of DEET in water was determined by gas chromatography with ion trap mass spectrometry. A sample was extracted in cyclohexane (4 mL samples: 4 mL cyclohexane). The separation, identification, and quantification of DEET were carried out using a Varian 450-GC gas chromatograph with 220-MS ion trap mass spectrometer and VF-5 ms (30 m × 0.25 mm) column (Varian, Inc., USA). A 1 *μ*L of sample aliquot extract was injected in splitless mode. The injector temperature was 250°C. The initial oven temperature was set at 50°C for 1 min, increased in a rate of 30°C min^−1^ to 130°C for 1 min, increased in a rate of 16°C min^−1^ to 230°C, held for 1 min, increased in a rate of 60°C min^−1^ to 280°C, and held for 1 min. Total run time was 13.75 min. Certified standard DEET was purchased from (Sigma Aldrich, Co.). All solvents were GC/MS-grade purity (Chromservis s.r.o., Czech Republic).

### 2.7. Histopathological Examination

Samples of liver, gills, cranial, and caudal kidney were removed from 5 fish in each group. They were fixed in 10% neutral formalin solution and subsequently stained with haematoxylin and eosin. Histological changes in samples were examined by light microscopy.

### 2.8. Statistical Analysis

Statistical analysis was performed using Unistat 5.6 software. A Shapiro-Wilk test was done for the normal distribution. The differences among test groups were assessed with the Tukey-HSD test. Immunological parameters were evaluated by the unpaired nonparametric Mann-Whitney test.

## 3. Results

During the experiment, the mortality of fish was not recorded in both control groups as well as in the tested concentrations.

### 3.1. Biometric Parameters

There were no changes in HSI and CF in fish exposed to all DEET concentrations compared to both control groups after 28 days of exposure (Table 2).

### 3.2. Haematological Profile

The DEET exposure did not affect WBC, MCHC, values of Hb, and PCV of experimental fish. A significant increase (*P* < 0.05) in RBC was observed in the concentration of 1 mg/L compared to both control groups (Table 3). Further, a significant (*P* < 0.05) decrease in MCV and a decrease (*P* < 0.05) in MCH were found in the 1 mg/L concentration compared to both control groups (Table 3).

WBC and differential white blood cells count were not affected by treatment (data not shown).

### 3.3. Biochemical Profile

The only change in biochemical profile of the experimental fish was in the decrease (*P* < 0.05) of TAG in the DEET concentration of 1 mg/L compared to the control groups. The other parameters including activity of butyrylcholinesterase were not affected (Table 4).

### 3.4. Immunological Parameters

**Figure 1:**
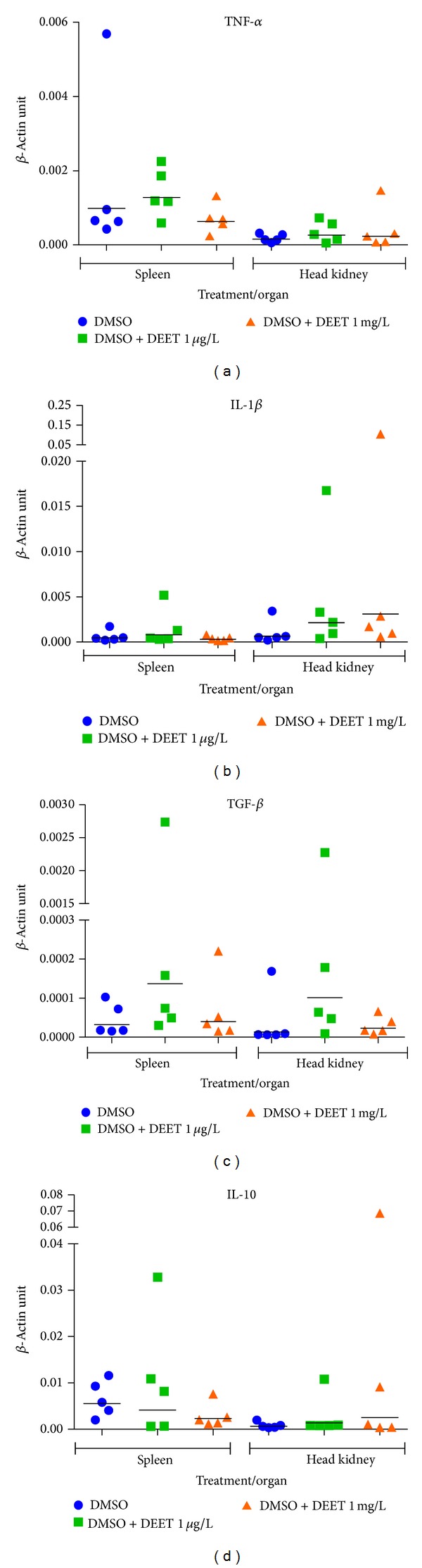
Graphs show individual values of the expression of cytokines versus housekeeping gene *β*-actin. Bars represent geometric mean values.

The exposure to DEET did not influence proinflammatory (TNF-*α* and IL-1*β*) and anti-inflammatory cytokine genes (TGF-*β* and IL-10) in any tested concentration of DEET (Figure 1).

### 3.5. Parameters of Oxidative Stress

Values of antioxidant enzymes activities (GR, GPx, GST, and CAT) and amount of TBARS are presented in tables for individual tissue (Tables 5, 6, 7, and 8). A significant (*P* < 0.01) increase in GPx was found in kidney in the exposure concentration of 1 mg/L compared to 1 *μ*g/L and a significant (*P* < 0.05) decrease in GPx was found in gills in the exposure concentration of 1 mg/L compared to the control group with DMSO. The catalase activity could be not determined in brain due to very low activity of this enzyme.

### 3.6. Histological Examination

A subchronic exposure to DEET did not cause marked specific histopathological changes in the DEET-treated fish.

## 4. Discussion

The amount of data on mechanism of action and chronic toxicity for DEET to aquatic environment is still limited. Acute toxic studies have found DEET to be slightly toxic for fish: 96 h LC_50_ for tilapia mossambica (*Oreochromis mossambicus*) and rainbow trout (*Oncorhynchus mykiss*) is 120–150 mg/L and 71.3 mg/L, respectively [31, 32]. Nevertheless our study has shown that even low concentration of DEET can influence red blood parameters of fish after 28 days of exposure. The increase in red blood cells in DEET concentration 1 mg/L indicates rise of erythropoiesis. Although the total amount of haemoglobin and haematocrit in blood was not changed, erythrocytes (MCV and MCH) decreased. Two-third decrease in mean corpuscular volume (MCV) of erythrocyte indicates a breakdown of erythropoiesis and a development of nonadequate erythrocytes. Higher occurrence of erythroblast was not recorded. In the study of dogs, a weak reduction of haemoglobin and haematocrit was noticed after 6 and 12 months of oral intake of DEET in concentration 400 mg/kg/day [33], but other red blood parameters were not affected. In adult fish, a spleen, the head kidney (pronephros), and mesonephros have been found to be sites of erythropoiesis [34]; specific histopathological changes of these organs in the DEET-treated fish were not noticed in our study.

The decrease in triacylglycerides in DEET concentration 1 mg/L was recorded. TAG are the most important energy-storing lipids and belong to major energy sources for the fish [35]. In this study, TAG decrease can indicate exhaustion of energy sources due to long-term stress.

Because DEET is reported to act as a neurotoxin through inhibition of cholinesterase [11], we concentrated on butyrylcholinesterase activity. However, butyrylcholinesterase was not affected. This finding supports results of studies about elevation of cholinesterase inhibition in insect only after common impact of DEET and cholinesterase-inhibiting insecticides [36, 37].

The immunological toxicity of DEET has not been extensively studied in fish before. Our observation was focused on the expression of proinflammatory (TNF-*α* and IL-1*β*) and anti-inflammatory cytokine genes (TGF-*β* and IL-10). There were not changes of the cytokine expression in head kidney and spleen in tested fish. Cytokines are the key initiator of immune reaction. They arise at the sites of entry of pathogens into organism; they stimulate inflammatory signals and thus the ability of resident and newly recruited phagocytes to eliminate the invading pathogens is regulated [38]. In teleostean fishes, such as carp and rainbow trout, the expression of interleukin 1*β* (IL-1*β*) mRNA can be stimulated by lipopolysaccharide alone or in combination with cortisol [39–41]. On the contrary, some toxic compounds as cyanotoxin anatoxin-a, for example, significantly inhibited proinflammatory (IL-1*β* and TNF-*α*) cytokines and induced anti-inflammatory (IL-10 and TGF-*β*) cytokines in common carp [42].

The effect of DEET on formation of oxidative stress was studied especially in insect [43] and rats [44–46]. Antioxidant enzymes, that is, GPx, GR, CAT, and SOD, keep the oxidative status in the cell. They reduce either free or membrane-bound hydroperoxides [47]. Glutathione S-transferase catalyzes the conjugation of the reduced form of glutathione to xenobiotic substrates for the purpose of detoxification [48]. In our study, we observed alterations only in case of GPx activities. The activity of GPx in kidney tissues increased in experimental group exposed to 1 mg/L of DEET compared to the DMSO control group. This tissue-specific GPx increase might indicate the adaptive approach by the fish to defend the oxidative stress [49]. Moreover, we also found decline in GPx activity in gill tissues of experimental group exposed to 1 mg/L of DEET compared to the DMSO control group. This alteration in GPx in gills might be due to the depletion of the enzyme. In fact, the fish gills were the first organ exposed to the toxic effluent [50].

## 5. Conclusions

Fish are an appropriate model for a further investigation of the biological effect of DEET on vertebrates due to its high frequency of occurrence in aquatic environments around the world. Although acute toxicity levels of DEET are high, low concentration after subchronic exposition can cause adverse effects on haematological parameters. To assess the effect of diethyltoluamide on the fish immune system, more immunological parameters need to be included in the future studies.

## Figures and Tables

**Table 1:** Primers used for gene expression by immunological examination of common carp.

Gene	Forward primer (5′-3′)	Reverse primer (5′-3′)
TNF-*α*	GCTGTCGCTTCACGCTCAA	CCTTGGAAGTGACATTTGCTTTT
IL-1*β*	AAGGAGGCCAGTGGCTCTGT	CCTGAAGAGGAGGCTGTCA
TGF-*β*	ACGCTTTATTCCCAACCAAA	GAAATCCTTGCTCTGCCTCA
IL-10	AAGGAGGCCAGTGGCTCTGT	CCTGAAGAAGAGGCTGTCA
*β*-actin	GCTATGTGGCTCTTGACTTCGA	CCGTCAGGCAGCTCATAGCT

**Table 2:** Biometric parameters in *C. carpio *for each tested group (*n* = 10).

Parameter	Control	Control with DMSO	DEET 1 µg/L	DEET 0.1 mg/L	DEET 1 mg/L
Body weight (g)	295.06 ± 40.76	267.33 ± 42.99	267.10 ± 44.88	273.17 ± 39.86	282.67 ± 46.26
CF	2.59 ± 0.19	2.59 ± 0.17	2.51 ± 0.21	2.59 ± 0.26	2.51 ± 0.14
HSI	1.87 ± 0.39	1.90 ± 0.26	1.79 ± 0.26	1.74 ± 0.27	1.81 ± 0.35

**Table 3:** Haematological values in *C. carpio *from control groups and groups exposed to DEET (mean ± SD, *n* = 10).

Indices	Control	Control with DMSO	DEET 1 µg/L	DEET 0.1 mg/L	DEET 1 mg/L
RBC (10^12^/L)	1.69 ± 0.35^a^	1.78 ± 0.41^a^	2.21 ± 0.74^a,b^	2.20 ± 0.62^a,b^	2.49 ± 0.42^b^
Hb (g/L)	74.05 ± 10.81	69.26 ± 13.93	73.56 ± 13.95	67.14 ± 8.31	71.70 ± 17.59
PCV (L/L)	0.26 ± 0.04	0.27 ± 0.03	0.26 ± 0.03	0.26 ± 0.02	0.26 ± 0.02
MCV (10^15^/L)	162.11 ± 34.65^a^	159.54 ± 34.23^a^	134.76 ± 52.87^a,b^	128.68 ± 41.65^a,b^	107.27 ± 18.43^b^
MCH (10^12^/L)	44.12 ± 11.73^a^	39.75 ± 8.36^a,b^	36.35 ± 11.51^a,b^	32.66 ± 10.30^a,b^	29.88 ± 9.31^b^
MCHC (g/L)	0.28 ± 0.04	0.25 ± 0.05	0.29 ± 0.11	0.25 ± 0.04	0.27 ± 0.07

Significant differences (*P* < 0.05) between groups are marked by different alphabetic superscripts.

**Table 4:** Biochemical indices in plasma of *C. carpio *from control groups andgroups exposed to DEET (mean ± SD, *n* = 10).

Indices	Control	Control with DMSO	DEET 1 µg/L	DEET 0.1 mg/L	DEET 1 mg/L
ALT (µkat/L)	0.70 ± 0.22	0.65 ± 0.11	0.88 ± 0.35	0.97 ± 0.36	0.67 ± 0.33
AST (µkat/L)	1.88 ± 0.69	1.69 ± 0.47	2.11 ± 0.39	1.95 ± 0.51	1.80 ± 0.52
ALP (µkat/L)	0.40 ± 0.21	0.54 ± 0.19	0.55 ± 0.22	0.52 ± 0.17	0.42 ± 0.17
Albumin (g/L)	10.94 ± 1.77	11.12 ± 1.57	10.89 ± 1.30	10.86 ± 1.22	10.92 ± 2.03
Total protein (g/L)	29.23 ± 2.12	27.67 ± 2.30	26.33 ± 3.02	27.26 ± 3.15	26.36 ± 2.52
Glucose (mmol/L)	3.46 ± 1.09	3.67 ± 1.18	3.24 ± 1.12	4.13 ± 1.39	2.93 ± 0.87
LDH (µkat/L)	5.67 ± 2.33	5.07 ± 0.73	4.45 ± 0.99	4.78 ± 1.61	4.15 ± 1.30
TAG (mmol/L)	2.37 ± 2.34^a^	2.12 ± 0.73^a,b^	2.21 ± 0.99^a,b^	2.15 ± 1.60^a,b^	1.88 ± 1.30^b^
Ammonium (mmol/L)	196.12 ± 66.35	186.6 ± 104.77	164.39 ± 37.74	156.70 ± 50.76	172.33 ± 73.20
Calcium (mmol/L)	2.14 ± 0.09	2.06 ± 0.20	2.06 ± 0.06	2.03 ± 0.31	2.06 ± 0.12
Phosphorus (mmol/L)	1.85 ± 0.35	1.84 ± 0.33	1.88 ± 0.22	2.01 ± 0.58	1.80 ± 0.26
Lactate (mmol/L)	2.91 ± 1.29	2.64 ± 2.48	2.11 ± 0.95	3.29 ± 2.17	2.85 ± 1.77
Cholesterol (mmol /L)	3.33 ± 0.47	3.12 ± 0.54	2.97 ± 0.41	3.07 ± 0.52	3.06 ± 0.63
FRAP (Fe^2+^ equivalent µmol/L)	559.76 ± 91.74	448.57 ± 104.53	506.90 ± 60.87	483.81 ± 52.25	465.98 ± 116.96
ButChE (µkat/L)	1.55 ± 0.67	1.22 ± 0.85	1.54 ± 0.75	1.66 ± 0.59	1.69 ± 0.53

Significant differences (*P* < 0.05) between groups are marked by different alphabetic superscripts.

**Table 5:** Antioxidant enzymes activities and amount of TBARS in liver of *C. carpio *in each group (mean ± SD, *n* = 10).

Parameter	Units	Control	Control with DMSO	DEET 1 µg/L	DEET 0.1 mg/L	DEET 1 mg/L
GR	(nmol NADPH/min/mg protein)	5.69 ± 0.98	5.03 ± 1.98	5.43 ± 1.51	4.79 ± 1.14	5.86 ± 1.06
GPx	(nmol NADPH/min/mg protein)	182.3 ± 61.5	203.9 ± 70.7	169.1 ± 50.3	203.8 ± 47.2	194.7 ± 39.3
GST	(nmol /min/mg protein)	260.6 ± 82.9	344.9 ± 110.3	310.0 ± 91.4	284.1 ± 97.3	319.6 ± 100.3
CAT	(µmol H_2_O_2_/min/mg protein)	365.7 ± 70.4	351.3 ± 67.9	332.2 ± 62.7	344.7 ± 56.3	332.1 ± 73.0
TBARS	(nmol/g sample)	36.4 ± 10.4	30.6 ± 6.6	35.8 ± 11.2	36.1 ± 12.1	32.7 ± 14.4

**Table 6:** Antioxidant enzymes activity and amount of TBARS in kidney of *C. carpio *in each tested group (mean ± SD, *n* = 10).

Parameter	Units	Control	Control with DMSO	DEET 1 µg/L	DEET 0.1 mg/L	DEET 1 mg/L
GR	(nmol NADPH/min/mg protein)	5.02 ± 2.23	4.41 ± 1.62	5.67 ± 2.41	4.98 ± 1.68	4.76 ± 2.26
GPx	(nmol NADPH/min/mg protein)	193.4 ± 33.1^a,b^	183.7 ± 45.7^b^	164.9 ± 39.4^a,b^	201.7 ± 29.8^a,b^	221.8 ± 31.4^a^
GST	(nmol/min/mg protein)	252.3 ± 43.7	275.4 ± 45.7	256.5 ± 55.9	265.2 ± 65.9	301.9 ± 65.1
CAT	(µmol H_2_O_2_/min/mg protein)	52.63 ± 8.60	42.90 ± 12.49	49.21 ± 15.98	42.06 ± 9.87	44.36 ± 17.40
TBARS	(nmol/g sample)	24.12 ± 4.53	25.06 ± 5.11	24.99 ± 4.66	26.27 ± 6.26	27.00 ± 8.85

Significant differences (*P* < 0.05) between groups are marked by different alphabetic superscripts.

**Table 7:** Antioxidant enzymes activity and amount of TBARS in brain of *C. carpio *in each tested group (mean ± SD, *n* = 10).

Parameter	Units	Control	Control with DMSO	DEET 1 µg/L	DEET 0.1 mg/L	DEET 1 mg/L
GR	(nmol NADPH/min/mg protein)	3.64 ± 0.49	3.94 ± 0.35	3.88 ± 0.59	4.33 ± 0.81	4.01 ± 0.62
GPx	(nmol NADPH/min/mg protein)	56.20 ± 6.76	60.96 ± 6.87	56.86 ± 7.30	58.33 ± 6.16	56.93 ± 3.97
GST	(nmol/min/mg protein)	212.30 ± 50.11	220.28 ± 53.98	263.46 ± 62.05	222.51 ± 40.33	231.73 ± 78,67
TBARS	(nmol/g sample)	9.09 ± 3.59	8.3 ± 2.23	8.92 ± 3.15	10.37 ± 4.50	10.02 ± 4.32

**Table 8:** Antioxidant enzymes activity and amount of TBARS in gills of *C. carpio *in each tested group (mean ± SD, *n* = 10).

Parameter	Units	Control	Control with DMSO	DEET 1 µg/L	DEET 0.1 mg/L	DEET 1 mg/L
GR	(nmol NADPH/min/mg protein)	6.38 ± 1.04	7.29 ± 1.37	6.93 ± 2.16	7.96 ± 0.96	6.35 ± 1.05
GPx	(nmol NADPH/min/mg protein)	43.67 ± 13.94^a,b^	54.87 ± 18.11^a^	43.09 ± 21.65^a,b^	37.31 ± 6.94^a,b^	34.28 ± 11.39^b^
GST	(nmol /min/mg protein)	116.9 ± 24.2	112.6 ± 36.1	104.7 ± 30.3	116.4 ± 33.2	110.1 ± 22.7
CAT	(µmol H_2_O_2_/min/mg protein)	10.12 ± 3.55	9.20 ± 2.61	10.40 ± 2.73	11.07 ± 2.44	10.88 ± 2.27
TBARS	(nmol/g sample)	39.76 ± 17.62	29.23 ± 8.87	31.01 ± 9.66	36.66 ± 14.03	31.86 ± 8.61

Significant differences (*P* < 0.05) between groups are marked by different alphabetic superscripts.
